# Improvement of the First Training for Baccalaureate Nursing Students –A Mutual Approach

**DOI:** 10.5539/gjhs.v7n7p79

**Published:** 2015-03-26

**Authors:** Marziyeh Asadizaker, Zhila Abedsaeedi, Heidarali Abedi, Hooshang Alijanirenani, Mehrnaz Moradi, Simin Jahani

**Affiliations:** 1 School of Nursing and Midwifery, Ahvaz Jundishapur University of Medical Sciences, Ahvaz, Iran; 2 School of Nursing & Midwifery, Shahid Beheshti Medical Sciences University, Tehran, Iran; 3 School of Nursing and Midwifery, Khorasgan (Isfahan) Branch, Islamic Azad University, Isfahan, Iran

**Keywords:** clinical practice, undergraduate, nursing student, action research

## Abstract

**Background:**

Examination of problems and application of strategies appropriate for clinical education and learning, especially nursing clinical principles and skills internship can improve educational process and satisfaction of nursing students. The aim of the current study was to revise the current status of the fundamentals of nursing course and implement an improvement plan (2012-2014).

**Participants & Methods:**

The present study reports the three rounds of a participatory action-research study with a mutual cooperation approach and focus group discussion, with participation of 104 stakeholders. Content analysis approach was used to analyze the data obtained in focus discussion interviews. In addition, evaluation and reflection were done during the operating rounds, with the participation of all members, including students, were involved. This research program was approved by Shahid Beheshti University of Medical Sciences in Tehran-capital of Iran, at the Research Deputy of Nursing and Midwifery School and ethics committee of the university.

**Results:**

The findings of qualitative study detected Lack of consistency in planning and implementation of curriculum, inadequate intra/extra-organizational communication management, inadequate student understanding of situation, improper control of restrictors and improper use of facilitators in teaching and in clinical setting, were among major challenges in clinical skills and principles internship process in the context of this study. Educational decision-making authorities of the School developed an operational program within national curriculum framework through cooperation and reflection in clinical skills and principles training program.

**Conclusion:**

Planning Fundamentals of Nursing training in partnership with all those involved in practice and education, together with students involved can be effective in reducing educational failures, gap between theory and practice, and in students’ accountability and satisfaction.

## 1. Introduction

Appropriate opportunities are needed for nurses to develop leadership skills and have greater decision-making authority, thus allowing frontline nurses to create innovative solutions to patient care issues (Robert Wood Johnson Foundation [RWJF], 2010). Clinical practice is a key component of Bachelor of Nursing education and is where students learn about nursing by having clinical experiences in different areas of practice (Fourie & McClelland, 2011).

Clinical work in nursing education is an important component of the nursing curriculum aimed at actively engaging student nurses with the necessary skills needed for the nursing profession. Clinical work provides the necessary practical skills that the students need (Awuah-Peasah, Akuamoah Sarfo, & Asamoah, 2013). In order to develop consistent evidence-based practices, concise research evidence and an operation model for standardizing practices are needed (Holopainen, Korhonen, Miettinen, Pelkonen, & Perala, 2010).

Since the Fundamentals of Nursing (FoN) course is the first professional course for nursing students, any failure during the course might negatively affect the students’ behavioral development process and be carried on to later years in the course and influence their interest, decisiveness, and opinions toward nursing as a profession (Karabulut & Ulusoy, 2008).

FoN introduce basic nursing principles, including concepts that form the theoretical basis for the role of Registered Professional Nurse (Touro College School of Health Sciences, 2013). The Fundamentals of Nursing course aims to teach basic theories, notions, principles, and methods. The course helps students to gain a good understanding about the place of the nursing profession in society, the relationship of nursing with other professions, and to appreciate nurses’ own duties, rights, and responsibilities (Karabulut & Ulusoy, 2008). The practice of clinical teaching differs somewhat from program to program. It is not possible to recommend a set of clinical teaching strategies that will be equally effective in every nursing education program. Rather, the faculty must make decisions about clinical teaching that are congruent with the planned curriculum and relevant to its context (Gaberson & Oermann, 2010).

A philosophical context for clinical teaching influences each individual’s perception toward the clinical teacher role and the process of teaching in clinical settings. This philosophy includes fundamental beliefs about the value of clinical education, teachers’ and learners’ roles and relationships, and how desired outcomes can be achieved (Gaberson & Oermann, 2010). In relation to this, Benner and co-workers believe that nursing education programs must be redesigned to prepare nurses for new responsibilities and challenges in these health care environments (Svejda, Goldberg, Belden, Potempa, & Calarco, 2012).

Current literature stresses the need for nurse educators to move away from the traditional approach of didactic teaching toward one that incorporates the facilitation of learning (Mower, 2010). Since 1930, most clinical teachings have been organized for a small group of students on one or more patients and headed by faculty members. Student nurses often provide care for patients in settings that appear alien to them (Niederhauser, MacIntyre, Garner, Teel & Muray, 2011). The majority of Iranian nursing schools, including the School of Nursing and Midwifery of Ahvaz University of Medical Sciences, also use clinical education in the conventional manner.

The nursing literatures confirm that educators should design strategies for more effective clinical teaching (Sharif & Masoumi, 2005). Recently, partnerships between schools of nursing and service settings in hospitals and other health care agencies have led nursing research and practice toward development of new approaches to improve clinical learning (Svejda et al, 2012). The present study aimed to review and improve the status of clinical education process in the Fundamentals of Nursing course.

## 2. Methods and Participants

This paper reports a partnership change in education process of first training for undergraduate nursing students. Action research with collaborative approach was used in this study and focus group discussion, and with participation of all stakeholders, including students. We investigated first training process, identified challenges and revised FoN training course as the first cornerstone in professional nursing in the School of Nursing and Midwifery of Ahvaz Jundishapur University of Medical Sciences in south west province (Khoozestan) of Iran. The present study was built on three cycles of action research. This research program was approved by Shahid Beheshti University of Medical Sciences in Tehran (Capital)-Iran, at the Research Deputy of Nursing and Midwifery School and ethics committee of the university. In the present action research study, focus groups discussion and content analysis were used for qualitative data collection and analysis of the current status of the undergraduate FoN clinical education. A purposive sampling method was used for the formation of five focus group sessions (6 to 10 people) and one interview of double persons for data collection and detection of challenges and also, six change planning sessions. Reflection and Evaluation were done during the operating rounds, with the participation of all members, including students, were involved.

### 2.1 Participants

One hundred and four stakeholders including faculty and clinical setting administrators, academic members, undergraduate students and nursing staff participated in this study (Table 1).

**Table 1:** Participations numbers according to position and research stages

Participants numbers*	Nurse & Head nurse	Clinical/education supervisor	Administrator of Nursing Services	Nurse Consultant of deputy treatment	Student	Head of education office	Faculty trainer	Head of fundamentals group	Director of Nursing group	Deputy Education Faculty	Chief of Faculty
Reconnaissance (42)	5	5	3	2	16	1	8	-	1	1	-
Planning (11)	-	2	1	-	-	1	4	1	1	1	-
Implementation, reflection & reform (66)	4	-	-	-	56	-	4	1	1	-	-
Qualitative final evaluation (18)	3	-	-	-	8	-	3	1	1	1	1

### 2.2 Setting

This research was conducted in nursing and midwifery school and collaborated with three educational hospitals affiliated to Ahvaz Jundishapur University Medical Sciences (Golestan, Imam Khumeini and Razi hospitals) in the south-west of Iran.

### 2.3 Action

For the focus discussion sessions, several questions were semi-structured and pre-prepared. Questions to be asked with respect to each group (students) were: How has your fundamental training course been? Describe your experience in fundamental training, from the first day until the last day. According to the responses, the next question asked was: What was the role of the teacher? What about staff? Instructors and clinical nurses were asked: How do you analyze the Fundamentals of Nursing training? Describe your experience in this regard. Describe your training program as a whole course. Describe your daily routine for clinical education. As the discussions proceeded, probing questions were used to elicit more in-depth responses about issues of interest that emerged. The duration of the interviews ranged from 75 to 105 minutes, according to participants’ circumstances. All interviews were recorded by MP3 voice recorder and transcribed verbatim.

### 2.4 Data Analysis

In this study, a qualitative content analysis was used based on the method proposed by Graneheim and Lundman (Graneheim & Lundman, 2004). The interviews were read through several times to obtain a sense of the whole. The text was condensed and then divided into meaning units. The condensed meaning units were then abstracted and labeled with a code. The various codes were compared based on differences and similarities and sorted into subcategories and categories that constituted the manifest content. The tentative categories were discussed by two researchers and revised as required. What differed between the two researchers was their judgment about what comprised familiar and unfamiliar sensations and actions. A process of reflection and discussion resulted in agreement about how to sort the codes. Finally, the underlying meaning, that is, the latent content of the categories was formulated into a theme.

### 2.5 Trustworthiness

In this study, trustworthiness was based on, Lincoln and Gub’s (1985) recommended criteria (Streubert & Carpenter, 2011). Credibility and confirmability were achieved by returning a summary of the interviews and emerged codes for five students, total faculty and two staff nurses for checking and confirming. In addition, as a member of the groups (lecturer) involved and long association with environments in the study, the first researcher helped understanding of the environment and to build participants’ trust. She was leader and coordinator of all focus groups interviews. Peer-checking was done by the authors and three doctoral nursing students which resulted in similar finding. Maximum variation of the sampling and triangulation data collection enhanced the data credibility and confirmability.

## 3. Results

The transcripts of interviews were manually analyzed with the help of software “MSWord” and “MAXQda” “version 10”. After comparing the categories and subcategories, four themes were extracted including: Coherent plan and planning, intra/extra-organizational communication management, Student’s understanding of situations, control of inhibitors and proper use of facilitators were among major (Table 2). After classification of problems regards to 4 themes, educational decision-making authorities of the School developed an operational program within national curriculum framework through cooperation and participation in clinical skills and principles training program”.

**Table 2:** Themes and categories derived from data analysis

Categories	Themes
○ Inappropriate clinical education content ○ Non-adherence to standards appropriate to conditions ○ Educational steps ○ Ambiguity in assessment of students ○ Lack of integration of programs and programming	Coherent plan and planning
○ Communicational factors ○ Trainers’ position and characteristics ○ Student’s characteristics and conditions ○ Clinical nurses’ position and characteristics	intra/extra-organizational communication management
○ Student’s perception of training and trainers ○ Student’s feelings and perception of discipline	Student’s understanding of situations
○ Inhibitors ○ Facilitators	control of inhibitors and proper use of facilitators

In the following section, we provide an overview of the Fundamental of Nursing course structure and then describe how the themes were extracted.

**Structure of Fundamentals of Nursing Course:**

This course is offered in the first year of the Bachelor of Nursing and includes theory, practical and clinical sections.

- The theory section is taught in a three-unit course by nursing faculty members in the school classroom and attended by all beginning students.
- After completing the theory, the practical section (simulation) is taught and practiced by faculty trainers in the school’s Skills Lab.
- Throughout their theoretical and practical training, students are divided into several groups and their first clinical practice experience is completed under the supervision of a member of the faculty as an instructor in a training module for 10 days in one clinical environments.
-
  **Figure 1:**
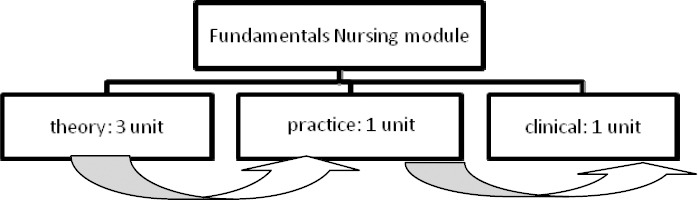
Fundamentals of Nursing course Structure

**1) Coherent plan and planning:**

The findings in this study suggest that an important aspect of clinical training within the fundamental nursing course is “integrated planning”. This theme emerged from combining categories labeled as curriculum content, standards appropriate to the circumstances, the stepwise and continuous training, insufficient coordination of program and planning and evaluation ambiguity. The following clarifies these concepts.

- Curriculum content huge: students should not be stressed by the educational content of their first clinical experience. Participants described the inappropriate nature of the clinical training content in the first clinical experience by novice students.
- Appropriate standards of the circumstances: Participants stressed maintaining the standards besides creating appropriate stress-free atmosphere. One faculty member said: “However, we must consider the situation of our society is somewhat different from Western standards. Therefore, we need to set our standards commensurate with our own community.” Participants stated that the structural changes needed to review clinical nursing tasks and procedures based on available educational standards.
- The stepwise and continuous training: In practical units, students are given practical training in various procedures. The student should be calm and proceed step-by-step to gradually become familiar with their position as a nurse and have the opportunity to adapt to the new situation and enter the clinical setting without extreme stress and frustration. Delivery of Fundamentals of Nursing principles and practice content and all instructions in a single 10-day intense training is impossible, and instead it should be taught on a continuous basis until the student graduates.
- Evaluation ambiguity: The evaluation component was one particular aspect with which the participating teachers and students had problems. According to the summaries of their opinions, it seemed that there was ambiguity in the evaluation criteria. Expectations and the evaluation criteria for students should be clear and appropriate to their learning ability and the training provided.

**2) Intra/extra-organizational communications management:**

Intra/extra organizational communications management was another theme derived from participants’ narratives. This theme was drawn from a combination of four categories: communication factors, workforce training providers (trainers), human learners (students), and clinical staff nurses.

**Relationship factors:** Proper coordination between the content of the theory and clinical practice was one of the important factors involved in human resource management associated with the Fundamentals of Nursing clinical teaching. Students felt the gap between theory and clinical training. On occasion, there was a mismatch between what nurses performed in clinical practice and what students learned in school, leading to the students’ confusion and uncertainty in clinical supervision. The difference between textbook learning, simulation practice in schools and clinical practice in a hospital is the current problem of coordination and communication. There is currently no controlled systematic communication between the school and educational hospitals.

**Educators:** First-year nursing students, who are learning their first clinical experiences, often face stressful days. Educational planning authorities are required to more carefully consider the selection of teachers and their mentors. Teachers and trainers must be mentally and physically fit and motivated, as well as interested in the nursing profession and be a positive role model for their learners. Participants believed that college instructors must be present in the clinical setting. Students need their instructors to play a supportive role— emotionally, educationally and clinically.

Some students felt safety in the presence of their instructors and wanted the direct supervision of instructors. Others saw them more as a reference for guidance and being available for students’ reference. One of the participants believed that the fundamentals instructors of theory, practice and clinic are considered by students as “role models” with all the behavioral, ethical, and scientific characteristics. Therefore, it is undeniable that professional instructors’ positive or negative features can lay the foundation for influencing future nurses. They can help reduce the gap between theoretical knowledge and practical clinical knowledge.

**Learners:** Students enter nursing for different reasons. Some of them have different interests, and nursing may not be appealing to them. Students’ interest rate needs to be surveyed by their instructor before training so they can be aware of their learners’ interests. This helps the teacher know how to treat them. Uninterested students are under more stress than others.

**Staff nurses:** Clinical nurses have very effective role on learning clinical skills and role modeling for students. They can be either positive or negative role models in this regard.

**3) Control of inhibitors and proper use of facilitators:**

One of the other themes in this study is “barriers and facilitators”. This theme emerged from the combination of categories: facilitating and limiting factors, and in turn the subcategories of clinical setting and school facilitators, and clinical setting and school limiting factors.

**Barriers:** The lack of accordance of skill lab practice to clinical performance; the lack of nursing care performed by many of the nurses in the wards; the students not being associated with staff nurses in primary care; failure to properly train in primary care given by the faculty instructor to the students; focusing on issues such as patient records review instead of patient care. If students are focused solely on the fundamentals procedures, this will not lead to the creation of student’s holistic view. The narratives of various students from various years that have experienced their first clinical practice showed that they felt the atmosphere and conditions governing the wards were inappropriate. Some of the causes posited include: the personal performance of trainer; patients requiring high traffic health care; in-fighting in the wards, and role of physicians in the ward. Sometimes stressful specialized wards were used for the first clinical experience environment such as neurosurgery and other special wards leading to extreme stress and confusion of students, suggesting the atmosphere in these wards was inappropriate for fundamental nursing learning. It was barely possible to undertake primary care due to an unfavorable atmosphere and the limitations of the equipment needed.

**Facilitators:** Integrated optimal physical space to practice (skill lab) in the school was one of strengths. Aspects proposed included: faculty members to coordinate with each other; creation of a more attractive role and better payment to recruit experienced trainers; recruiting and preparing clinical staff for students’ training; using experienced head nurses to enhance the clinical skills of less experienced trainers; integrating the steps for clinical training and daily schedule by all trainers and student groups to facilitate the learning process, and authorities’ willingness for more interactions between nursing office and trainers to reduce the theoretical-practical gap.

**4) Student’s understanding of situations:**

Student participants described their own sense and understanding of the professional tasks of nursing, trainers, and clinical practice amidst the learning experiences. The theme that emerged from the categories: “students’ perceptions of education and training” and “feelings and perceptions of students about profession” and in turn the subcategories: “student’s perceptions and feelings of professional nursing duties”; “student’s perceptions and feelings towards teaching practice”; and “student’s feelings toward trainer” were created.

- **The relative satisfaction of students:** Students’ feelings reflected a more positive perception of their mental training before going to their first clinical experience. Of course, some of them expressed relatively good experiences, but not great starts and sometimes difficulties while some described satisfaction with their training due to their ability to carry out the blood sampling and intramuscular injections.
- **Student’s feelings toward the teacher:** Students showed different feelings about their trainers in the workplace, such as they felt that instructors’ expectations of their students were disproportionately high and their understanding of the attitudes and practices of learning students was inadequate. Some students had positive opinions about teaching of the theory and practice in the skills laboratory but some others were complaining about large lesson topics and lack of facilities.
- **Student’s perceptions of the profession of nursing and tasks:** Students experienced confusion due to lack of adequate understanding of the responsibilities of the nursing student. For example, some of them believed the primary care is not nursing students’ duty.

**Current Status of Fundamentals of Nursing training Process:**

Regard to 4 themes remerged, the main challenges in FoN were included:

- Lack of integration in planning and implementing curriculum fundamentals
- Inadequate staffing and managing internal and external communications
- Lack of understanding of student’s status
- Lack of proper use of facilitators and insufficient control of barriers

**The operational plan to improve fundamentals of nursing clinical education:**

Data analysis and constant comparison continued for about eight months and continues during the study. The operational plan for clinical training fundamentals considering qualitative data derived themes are (goals) as follows:

G1: Integrated program
G2: Proper management of human resources
G3: attention to student’s perceptions and feelings

**Operational planning:**

Operational planning was conducted based on the comments and interviews with planners and stakeholders according to their different requirements, training and executive authority. Objectives were developed based on the problems list related to the fundamentals teaching that was taken from focus groups sessions. Planning groups were formed to identify strategic steps to achieve goals. Group members were selected based on their authority domain, experience and ability. Six planning sessions were organized by the Educational Deputy, and Director of Nursing and other clinically relevant authorities and researchers in the School of Nursing and Midwifery (Table 3 & Table 4). All sessions were led by the researcher.

**Table 3:** Detected Objectives (O) in related to each Goal (G) to improve training process of Fundamentals of Nursing

Goals	Objectives
**G1: Integrated program:**	**O1.** Organizing of FON Department **O2.** Enhance of the fitness of a number of students and instructors **O3.** Modification of the content of clinical training **O4.** Determine the proportional training steps with instructional context **O5**. Clarification of expectations and evaluation criteria
**G2. Proper management of intra/extra organizational communications**	**O1.** Enhance links between school and clinical setting (both partnerships in training). **O2.** Enhance relationship between Department of Fundamental of Nursing director and the Nursing Offices of hospitals. **O3.** Enhance relationship between trainer and Nursing offices. **O4.** Enhance communication between Instructor and head nurse. **O5.** Enhance empowerment and increased participation among clinical nurses in education of students.
**G3. Attention to student perceptions and feelings**	**O1.** Enhance student understanding of the educational process before entering the clinical environment.

**Table 4:** Action plan to improve educational process of clinical fundamentals of nursing

Objectives	Action plan
**Organizing principles and techniques group**	Selection of technical group head by trainers involved in teaching nursing principles and skills, and formal notification from education deputy Call for school teachers to cooperate in teaching of clinical principles and skills Assignment of permanent teachers of techniques theory and practice Allocation of appropriate time for technical group
**Improving student/teacher ratio**	Dividing practice student groups (more groups than before, and fewer students per group) Reducing student numbers in each group to 7
**Modifying clinical education content minimums**	Modifying minimums and training content of nursing principles and skills Compiling a summary of general nursing terms and abbreviations
**Appropriate formulation of educational steps**	Modifying educational steps of principles and techniques training
**Clarifying expectations and evaluation criteria**	Modifying student assessment form in nursing principles and techniques training
**Appropriate use of logbook in principles and techniques training**	Development of a draft logbook of nursing principles and skills training by a trainer and its use after modification and approval by other trainers
**Enhancing teaching and clinical relationship (collaboration)** **Improving technical group management communication with nursing office** **Improving trainers’ relationship with nursing office** **Improving trainers’ relationship with head nurse** **Improving capability and participation of clinical nursing in student education** **Improving capability of clinical experience of school trainer**	Informing teaching hospitals nursing managers of educational content of nursing principles and skills training and implementing their views in this area Assigning appropriate clinical wards for technical training, through coordination with nursing office Assigning mediating clinical nurses by nursing office as training assistants Preparing mediating nurses for education courses by the school through coordination with nursing offices Organizing educational and clinical coordination meetings before training, early in each academic term, with prior information and coordination of head of technical group

**Figure 2:**
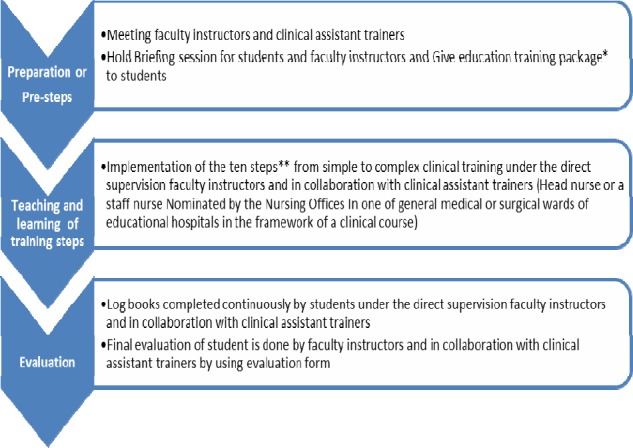
The operational model of Fundamentals of Nursing training process based on professional participation * Educational package included: lesson plan, must learning list, necessary terms and abbreviations, NANDA list, patient rights, ethics professional codes of nursing, log books and evaluation sheet. ** Refer to Table 5.

In order to perform and reform of an innovative plan, it was considered and reflected in three operational cycles. With the performance of this investigation and regards to current status, we recognized a local Operational Model as participatory teaching strategy. The model was developed in three operational cycles (figure 2).

**Initial Planning of reform:**

Six sessions were held with planning groups to determine action plan to achieve goals, given area of members’ authority and their experiences and capabilities. Meetings were held with coordination of educational deputy of Midwifery and Nursing School and Nursing Group Manager and other clinical and research beneficiaries. Action plan and goals were developed, considering the categorized problems in the process of training Fundamentals of Nursing. All academic planning in the country is performed centrally. Changes implemented in the training program should also be within the academic curriculum framework. Thus, given the inhibitors and facilitators in the context of study, training program was modified and adjusted. The researcher conducted all meetings of planning “Collaborative Adjusted Clinical Training” (CACT).

**Implementation, reflection and evaluation phase:**

After examining problems and solutions, an “initial operational model of Fundamentals of Nursing training process based on professional participation” was developed and implemented in accordance with participants’ decisions. Given the proposed program, “briefing meeting” and “stress management workshop” were held for students before entering clinical setting and a day before training. This program was performed by a member of School of Psychiatry, with attendance of all students involved and Head of clinical principles and skills group. In this meeting, students were provided with an educational package, which included: lesson plan, educational goals and steps, evaluation sheet, Patient’s Rights Charter, Logbook, and Nursing Diagnosis list (NANDA).

In each operational cycle, Process of training program was assessed and reflected in action during 0f 10 (equal one course unite) days nursing fundamentals training, so that attempts were made to resolve some of the problems during implementation. By the end of training period, educational process was evaluated by the trainer, head-nurses and clinical nurses assisting trainer and involved in the study in qualitative form (conversation, observation, and assessment sessions) and training students in qualitative form (short personal and group interviews, observation, and participation in assessment sessions).

The following are examples of formative assessment and reflection in one of the operational cycle:

**Positive points:**

- Coherence in planning
- Communication and interaction among faculty trainers, exchange of views from start of training, and attending evaluation meetings
- Interaction among trainers and head-nurses as trainer-assistant and educational planners
- Cooperation and welcoming of nursing office in hospitals under study in affecting changes and modifications through introduction of a nurse or head nurses in principles and techniques training wards as training assistants, and continued cooperation during the first cycle
- Cooperation of head-nurses and clinical nurses: accountability of nurses and making facilities available to technical students by nurses and head-nurses of internal and surgical wards in Golestan hospital were satisfactory. The internal ward trainer stated that “we used to have trainees in this ward before, but cooperation of personnel … (head nurse-training assistant) is much better now, and I feel much more comfortable and better now than before.”
- Trainer, students, and head-nurse satisfaction with training assistant in surgical ward in Imam Hospital, where training assistant was an enthusiastic young nurse of day shifts. The head-nurse of the same ward stated: “It is Ok, better, the difference with before is that now Miss F (training assistant) supervises. Duties are less neglected, and their trainer also controls things. It is much better than before, and jobs are more coherent, so is their program. Before, it used to be all over the place, and when students left, we had a lot of problems, but now it is not like that anymore. Both their trainer and Miss F monitor everything. Sometime Miss F is so busy, and I ask her if she has finished with us, and she says, they will become our colleagues in the future, if we do not work with them, then we will have problems later, students are more responsible.” The head nurse was generally happy, and rated communication between students, trainer, and the ward positively.
- Students had very positive evaluation of performing hospital round program on the first training day, conducted with their trainer (more or less), which is referred to as Round the Hospital (RTH) in this study.
- Recording students’ activities in the logbook.

**Weak points:**

- Students’ dissatisfaction with quality of “stress management workshop” before training
- Lack of uniformity of educational steps in all three technical training wards on determined days, given students and trainers’ statements. Reasons should be stated and require rearrangement in their order.
- Performing the first step in the surgical ward in Golestan Hospital (student familiarity with hospital and nursing hierarchy) was limited to that particular ward only. [This shortcoming was compensated in the second round by one of the trainers.]
- In Golestan surgical ward, communication between trainer and head-nurse (as training assistant) is not felt, and the trainer does not feel the need for head-nurse’s help in students’ clinical training.
- Head-nurses as trainers, because of busy schedule, did not much participate in students’ training and communication.

**Modifications required:**

Flexibility in implementation of intermediate (days 4-7) educational steps. Thus order of steps was altered in the following form:

1). On the first day of training, trainers should act in a coordinated way to familiarize students with physical environment of hospital and different wards, as well as nursing hierarchy (matrons to supervisors and different ranks in hospital rounds)
2). Explanation of educational steps and goals for students by trainers; communication with patients, TPR charts, and file sheets
3). Begin process of nursing with taking patient description, in addition to previous cases
4). IV and solution therapy care

The 4, 5, 6, and 7 steps are flexible depending on the ward conditions. Steps 8, 9, and 10 remain as before.

- Hospital round, as the first training step, should be performed by technique trainers for trainees in initial groups who did not receive first round.
- In performing steps, in all three wards, trainers should be more coordinated
- Given students’ dissatisfaction with stress management workshop, and their proposal to hold briefing meeting by technique trainers before training (there was no chance to perform the second round, and was postponed to the third round)

By the end of training period, educational process was evaluated by the trainer, head-nurses and clinical nurses assisting trainer and involved students in the change step, and in qualitative manner (conversation, observation, and assessment sessions). By the end of three cycles, in presence of educational planning authorities and representatives of students involved, the final evaluation was performed and local operational model was modified and adjusted (figure 2, table 5).

**Table 5:** Training steps of Fundamentals of Nursing according to its training days

Training Days/Training steps	
**1**	- Hospital and ward Round and orientation - Understanding student tasks and infection control
**2**	-Communicate with the patients regarding ethics (code of nursing ethics and patient rights), -- Safety education, monitoring and charting vital signs - Familiar with patient kardex and record and its terms and abbreviations
**3**	- Primary care regard to client needs (change sheet, mouthwash, bath in bed, etc), learning how to move patients (out of the bed, changing position) - Taking nursing history
**4**	- IV & serum therapy care - Control and record of fluid intake and output.
**5**	- Oxygen therapy, deep breathing and effective coughing and wound dressings care
**6**	- Meet nutritional and elimination needs, NGT care, perform gavage and Foley care.
**7**	- Familiarity with 6 correct medication administration - pharmaceutical calculations - Drug preparation (drag injectable drugs, oral drug preparation)
**8**	- Report writing - Preparation of training materials for the non-invasive tests and procedures.
**9**	- Monitoring and evaluation of student work
**10**	- Monitoring and evaluation of student work

* Note: Steps 4, 5, 6 and 7, depending on the ward situation are flexible and can be moved.

After final evaluation, positive points and modification needed in innovative educational model were detected.

**Positive points:**

○ Group cohesiveness and Highlight the role of nursing clinical principles and techniques [FoN]
○ Improvement of inter personal communications within education setting (nursing school)
○ Improvement of instructors between practitioners communications
○ Satisfaction of holding a briefing in the first cycle and improve it by the faculty instructors in the third cycle (according to surveys of students participating)
○ Support and participation of supervisors and matron of the hospitals studied, in the first step in implementing training “round the hospital”.
○ Stabilizing the role of assistant trainer (head nurse or nurse appointed by the Nursing Office manager)
○ Record the activities of students learning in log books
○ Educational authorities report on readiness to continue the implementation of collaborative process in the next semester educational
○ Reduce and adjusted number of students per instructor
○ The coordination of instructors in collaborative of training process
○ Educators satisfaction with the collaborative educational process
○ Improvement and integration of educational steps
○ student satisfaction with the overall of Internship and collaborative training process and their accountability in the education and care settings

**Modifications needed:**

- Need to improvement of quality performance of “briefing session”
- Head nurses, “assistant trainers” devoted more time to teach
- More attention in filling the log books
- Modified training evaluation sheet

## 4. Discussion

In action research studies, the focus of change was mainly organizational, practice development or education. The methods of choice are usually qualitative, and designs are pluralistic in this respect. In the present study, we used participatory action research method for identifying and enhancement of current process of first nursing training. This may help policy makers to decide about the most effective and efficient interventions (Changiz Malekpour & Zargham-Boroujeni, 2012). This study aimed to determine, review and improve the status of the clinical fundamental nursing teaching of undergraduate students. The findings in this study suggest that an important aspect of clinical training for Fundamentals of Nursing is integrating planning and scheduling. Appropriate content for teaching theory and clinical practice and continuing education, emphasizing maintaining the necessary standards and minimum training standards proportional to different conditions and contexts, are required. Yang et al. (2013) studied the first clinical experience of first-year nursing students. Themes were extracted related to nursing education program effectiveness in this study such as: continuing education or “endless learning” and “integration”, which confirm our study in these aspects.

Moreover, expectations and evaluation criteria should be clear and appropriate to each student’s ability to learn and how to train him/her. Awuah-Peasah and Co-workers (2013) wrote that nursing students should be familiar with what is expected of them before entering the training environment. Oermann and Gaberson confirm that the student is a learner, not a nurse, so their preparation should be tailored to their expectations. Some teachers and nurses are expected to appear as an expert without having enough time to practice and refine their performance (Oermann & Gaberson, 2009).

As Sharif and Masoumi (2005) stated, not being aware of differences between actual and expected behavior in the clinical setting creates conflicts in nursing students. Nursing students receive instructions that are different from what they have been taught in the classroom. Students feel anxious and this anxiety affects their performance. Nursing students clearly identified that the initial clinical experience was very stressful for them (Sharif & Masoumi, 2005).

Conducting any program is not possible without having competent and qualified human resources. In addition to present expertise and experienced human resources, professional inter-organizational and intra-organizational relationships are also important. Nursing staff workload and workday structure, as well as professional development, also have this problem. Thus, students felt that they could not benefit from nurses’ skills, but they did benefit when nurses were patient with them. In many clinical centers, it is difficult to release staff to participate in clinical education, but collaborative strategies can reduce the negative attitudes of nurses toward nursing students in the clinical learning environments (Hathorn, Machtmes, & Tillman, 2009).

Clinical education experience is personal and interpersonal, and has specific rules and regulations that require the active participation of instructors and students (Karimi Moonaghy, Dabbaghi, Oskouie, Vehviläinen-Julkunen & Binaghi, 2010). Collaborative models of clinical education increase confidence and competence in clinical care because of teaching expectations and the mentors’ expectations of their students’ integration (Svejda et al, 2012). Strategies include intensive inter professional collaborations and radical curriculum revisions. Nurse Educators must work with all stakeholders to create effective and lasting change (Fitzgerald, Kantrowitz-Gordon, & Katz (2012).

Improving educational capacity through nursing faculty development has been proposed as one of several strategies to address a complex health human resource situation (Evans et al, 2013). Committed partnerships across professions are necessary for successful integration of inter professional education. Through these endeavors, nurses can achieve their full practice potential (Abbott, Fuji, & Galt, 2013).

In the traditional model, clinical nurses are less involved and teachers and students are seen as guests of the clinical venue; in a collaborative model, support by nursing staff has led to student satisfaction, confidence in patient assessment, communication skills and more responsibility for their learning. Nursing staff have also acknowledged that students are more responsive toward patient care (Fourie & McClelland, 2011).

Another theme that emerged in this research was participant students’ perceptions and feelings. This theme emerged from combining categories: “Student’s perceptions about trainer and training” and “Student’s perceptions about the discipline and profession.” Students had a more positive perception before going into training environment and sometimes describe their first clinical experience as stormy. Students showed different feelings toward their trainers. Some felt supported with the trainer’s presence and others experienced stress. Sometimes they were faced with a reality that their perceptions do not comply with reality and they also felt that their teachers did not understand them.

Results from Lambert and Glacken’s (2005) study showed that nursing students are not compatible with clinical practices and they are likely to experience difficult learning processes. Orientation programs design for faculty need to be realistic, with supportive systems and interventional programs to strengthen nursing students’ competence. Staff nurses and lecturer practitioners play key roles by being influential in creating and sustaining this supportive clinical learning environment (Lambert & Glacken, 2005).

Yang’s study (2013) aimed to explore and understand the meanings and structures of the first clinical experience and suggested that awareness of the clinical learning experiences leads to useful insights for faculty involved in teaching nursing (Yang, 2013).

In this study, the participants pointed out various restrictions such as faculty shortage, and work overload of staff nurses. On the process of optimal clinical education, they also noted some instances that could be used as facilitators in the process of appropriate fundamental nursing training such as enhancing links between school and clinical setting including relationships between Department of Fundamental Nursing director and the Nursing Offices of hospitals, Trainer and Nursing Offices, Instructor and head nurse; as well as empowerment and increased participation of staff nurses for training of students. Another recommendation was determining the clinical liaison nurses as assistant trainers by the Nursing Offices and provision for the cost of their work and teaching certificate. Fourie and McClelland (2011) note that tracking costs and sufficient funds are made to determine a more structured approach and supportive environment to students before the start of a partnership model (Fourie & McClelland, 2011).

## 5. Conclusion

Participation of all beneficiaries in all planning stages of the first nursing training process as cornerstone of professional clinical operations within existing educational curriculum framework can be effective in reducing educational failures, gap between theory and practice, and in students’ satisfaction. Our findings, suggested the nurse and other clinical researcher to use a mutual approach to reduce the gap between theory and practice in addition to the technical approach and test the theory. This approach is more democratic and the participants feel that they actually contribute to change and improve up their systems. Therefore, they are more committed to the process and follow up change.
